# Heart Valve Biomechanics: The Frontiers of Modeling Modalities and the Expansive Capabilities of *Ex Vivo* Heart Simulation

**DOI:** 10.3389/fcvm.2021.673689

**Published:** 2021-07-08

**Authors:** Matthew H. Park, Yuanjia Zhu, Annabel M. Imbrie-Moore, Hanjay Wang, Mateo Marin-Cuartas, Michael J. Paulsen, Y. Joseph Woo

**Affiliations:** ^1^Department of Cardiothoracic Surgery, Stanford University, Stanford, CA, United States; ^2^Department of Mechanical Engineering, Stanford University, Stanford, CA, United States; ^3^Department of Bioengineering, Stanford University, Stanford, CA, United States; ^4^University Department of Cardiac Surgery, Leipzig Heart Center, Leipzig, Germany

**Keywords:** heart valve biomechanics, modeling modalities, heart simulation, clinical translation, surgical therapies

## Abstract

The field of heart valve biomechanics is a rapidly expanding, highly clinically relevant area of research. While most valvular pathologies are rooted in biomechanical changes, the technologies for studying these pathologies and identifying treatments have largely been limited. Nonetheless, significant advancements are underway to better understand the biomechanics of heart valves, pathologies, and interventional therapeutics, and these advancements have largely been driven by crucial *in silico, ex vivo*, and *in vivo* modeling technologies. These modalities represent cutting-edge abilities for generating novel insights regarding native, disease, and repair physiologies, and each has unique advantages and limitations for advancing study in this field. In particular, novel *ex vivo* modeling technologies represent an especially promising class of translatable research that leverages the advantages from both *in silico* and *in vivo* modeling to provide deep quantitative and qualitative insights on valvular biomechanics. The frontiers of this work are being discovered by innovative research groups that have used creative, interdisciplinary approaches toward recapitulating *in vivo* physiology, changing the landscape of clinical understanding and practice for cardiovascular surgery and medicine.

## Introduction

Valvular heart disease is a significant cause of global morbidity and mortality, with a prognosis rivaling many types of cancer (1). While there are a wide variety of repair operations and devices, most of these strategies have been historically based upon anatomy and subjective visual appearance, and quantitative mechanical foundations have been limited as interventional insights have largely been driven by clinical outcomes. Moreover, many valvular pathologies are rooted in biomechanical changes, yet the technologies for studying these pathologies and identifying treatments have largely been limited. This disconnect is particularly evident in the lack of surgeon consensus regarding debates such as optimal repair techniques and mechanisms behind disease pathologies and operations (2–5). However, significant advancements have been made to better understand heart valves and have largely been driven by *in silico, ex vivo*, and *in vivo*

modeling technologies, which have provided the underlying platforms for generating many new analyses and insights. In particular, *ex vivo* modeling represents an especially promising class of translatable research that leverages the advantages from both *in silico* and *in vivo* modeling to provide deep quantitative and qualitative understandings of valvular biomechanics. Each of these modalities has unique advantages and disadvantages, and presented is a review of recent, impactful developments in the field, including the key outlooks and limitations of the technologies.

## *In Silico* Modeling

Computational simulations have been a major driving force for generating greater intuition of heart valve mechanics. As imaging modalities and mechanical characterization of biological systems have improved, so have the relevance and realism of these models to better predict physiologic outcomes. A particular area of study that has seen major advancements from *in silico*, image-based modeling is that of MV dynamics. Pioneering the clinical application of new computational technologies, researchers have used real-time 3D echocardiography to generate patient-specific computational models of the MV (6–11). Using these models, publications have revealed pathological dynamics and generated useful quantifications based on valvular geometry. For example, by characterizing mitral annulus motion, researchers found that the cyclic, saddle-shape conformational changes of the annulus are important for efficient operation of the valve, reducing systolic strains on the posterior leaflet and peak leaflet stresses, potentially improving long-term durability (12, 13). These findings have large implications for informing clinical practice, as the annuloplasty procedure, which often results in fixing the mitral annulus, is the most common MV surgical intervention (6, 8). By correlating native mechanics to pathological annular biomechanical changes *via* image-based computational modeling, these studies have compared annuloplasty rings and identified areas of surgical intervention for improving care. Additional studies that model the MV have provided valuable insights describing the physiologic, pathologic, and repaired operation of the valve (14–21).

The study of prosthetic valvular replacements, particularly of the AV, is another area where *in silico* models are making great strides. Using computational flow or imaging-based models, researchers have created unique metrics that evaluate valvular replacement performance, addressing aspects such as leaflet thrombosis, abnormal flow patterns, blood damage, hinge design outcomes, paravalvular leakage, and left ventricular dynamics, while also creating novel preoperative, patient-specific planning protocols and prediction tools for surgical or transcatheter interventions (22–32). These models provide detailed views through unique quantifications, developing deeper intuition behind the valvular mechanics. They have been crucial for understanding complex phenomena, such as the value of a bi-leaflet prosthetic or the effect of neosinuses on the downstream aortic wall shear stresses and turbulence, and are providing useful new perspectives for comparing treatment options (22–24, 33).

Specifically regarding the AV, *in silico* modeling has enabled the simulation and study of many relevant mechanical parameters, leading to a detailed, new understanding of the native physiology as well as complex pathologies. Many innovative models have been developed and utilized to answer questions regarding, for example, hemodynamic anomalies and surgical repair techniques (34–36). In particular, a series of computational studies aimed at researching the bicuspid AV pathology has elucidated new insights such as the phenomena that specific bicuspid AV geometries induce regions of high stress concentrations in the leaflets, which may influence the mechanics of long-term degradation as well as leaflet calcification (37–39). These studies, coupled with hemodynamic simulations that can identify the temporal effects of blood flow on the AV leaflets, have generated powerful tools for comparing healthy and structural disease states with computationally generated and, in some cases, patient-specific models (40–42). This work highlights the power of *in silico* modeling: creating new connections between phenomena that would otherwise be unintuitive to understand and difficult to detect and setting a new benchmark of material and actionable mechanical data.

*In silico* models have many unique advantages for studying the heart. Specifically, by using computational models, researchers have generated unique comparisons, visualizations, and quantifications that would otherwise be very difficult to do. For example, using a lattice-Boltzmann method, a recent publication outlined the dynamics and surface shear stresses on a specific prosthetic valve hinge at an individual blood platelet level of granularity, generating a blood damage index based on a linear shear stress-exposure time model (23). Such metrics are almost impossible to quantify without computational modeling. However, *in silico* models require many unifying, homogenous assumptions, such as cellular response, parameter identification of tissue properties, and muscle fiber interactions, which can be quite far from the heterogeneous, patient-specific, *in vivo* physiology (43–45). While work is being done to incorporate neural networks for building accelerated, enhanced models, their unifying assumptions are often obstacles for generating accurate, relevant conclusions (46, 47). Regardless, *in silico* modeling provides a truly unique value proposition for greater biomechanical intuition of the heart, and these studies demonstrate the foundational research that has guided further experimentation and, ultimately, clinical practice.

## *Ex vivo* Modeling

**Figure 1:**
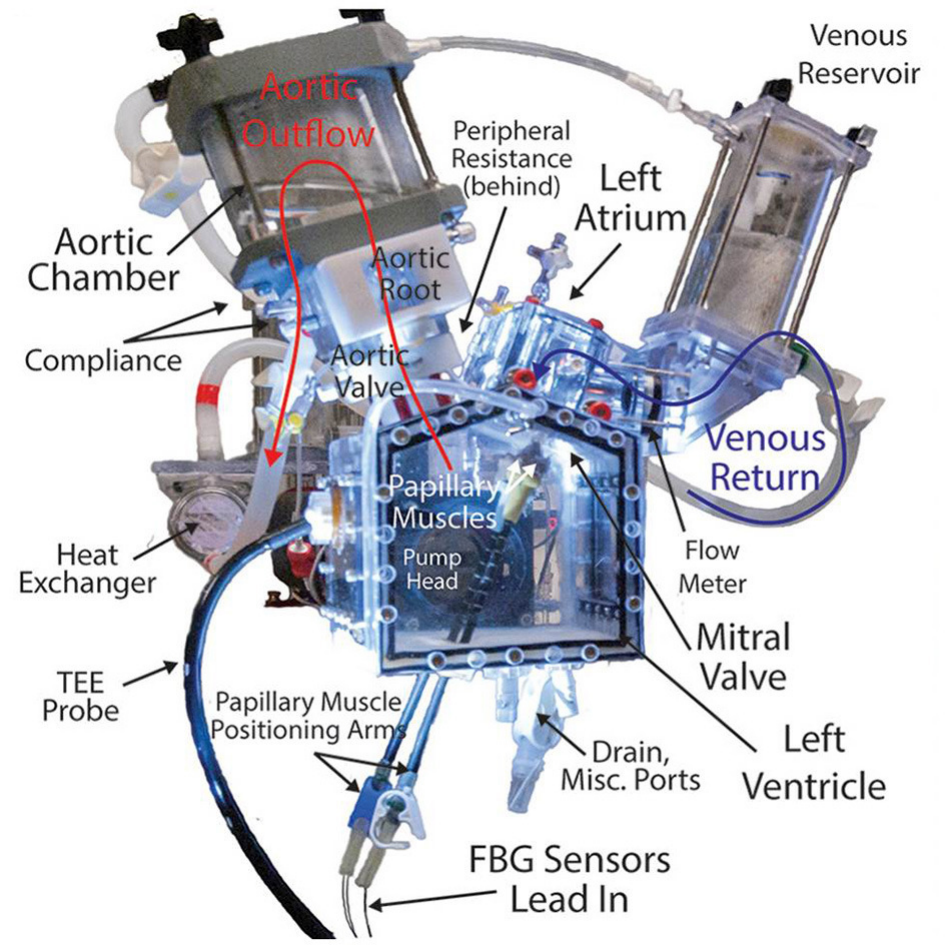
Detailed, labeled image of a left heart simulator. This system uses explanted large animal heart valves and a piston pump to generate and measure physiologic pressures and flows through the valves to study disease pathologies, repair techniques, and surgical devices. Simulators such as these provide useful platforms for modeling heart valve biomechanics and represent a promising new class of research in this field, directly informing clinical understandings and practice.

*Ex vivo* heart simulators allow researchers to study heart biomechanics with a high level of accuracy and control and are powerful tools for better understanding native physiology, pathologies, and treatments. This modality usually involves mounting explanted large animal valves in fluid filled chambers and/or flow loops, driving physiologic flows with a pulsatile linear piston pump (Figure 1). These simulators are often instrumented with flow and pressure sensors to measure hemodynamics, and valves can be imaged using echocardiography, videography, or MRI. Moreover, many of these systems have leveraged the use of a symbiotic, multimodal approach with computational strategies, which have provided critical biomechanical insights, particularly regarding deeper hemodynamic understanding, using technology such as particle image velocimetry (48–52). This comprehensive modeling approach has allowed for the use of more sophisticated computationally derived data, which when applied to *ex vivo* systems, provides more detailed quantitative analyses.

Much of the early work of *ex vivo* modeling focused on mitral, aortic, and tricuspid valvular physiology and biomechanics (50, 53–60). Building on this technology, researchers have studied a wide variety of phenomena including coaptation area, mitral and tricuspid annular geometry, mechanisms behind regurgitation, and transcatheter prosthetic valve design and deployment strategies (61–78). Many of these publications have changed the understandings of key interactions, particularly those of MV annulus dynamics and the downstream effects on MR and annuloplasty repair. In a series of studies, researchers decoupled the effects of the mitral annulus and leaflet geometry, demonstrating that the D-shaped annulus allows for more efficient LV filling by minimizing energy dissipation (58, 61, 79, 80). Extrapolating further, a recent publication focused on creating a dynamically contracting mitral annulus to recapitulate the natural annular changes. With stereophotogrammetry, the authors tracked the anterior leaflet of a porcine MV, identifying increased leaflet strain when implanting a rigid annuloplasty ring (81).

Novel research is expanding the capabilities and applications of *ex vivo* modeling, focusing on generating immediately, clinically translatable insights for valvular treatment and surgical care. In a series of recent studies, researchers evaluated the biomechanical effects of apical and papillary neochord anchoring locations for MR repair, exploring the mechanical outcomes of novel percutaneous neochordoplasty technologies (82, 83). These studies were enabled by the development of optical fiber-based force-sensing neochordae, which provide minimally invasive, highly sensitive chordal strain measurements (84). It was determined that apical neochord anchoring increases the rate of loading on the surrounding chordae and neochord, suggesting increased stresses on and long-term durability concerns for the MV apparatus (83). This work was directly relevant for percutaneous neochordoplasty technologies, as the results provided mechanical bases that possibly corroborated early clinical results. A follow-up study proposed a new device design to alleviate these increased loading rates by introducing an elastic transapical artificial papillary muscle that shortens the length of the chord and dissipates sharp increases in force (85). Studies such as these demonstrate the transformative nature of *ex vivo* simulation, altering the clinical landscape by providing convincing new evidences for informing surgical and interventional care, which are driving how treatments are being developed and evaluated (86–90).

**Figure 2:**
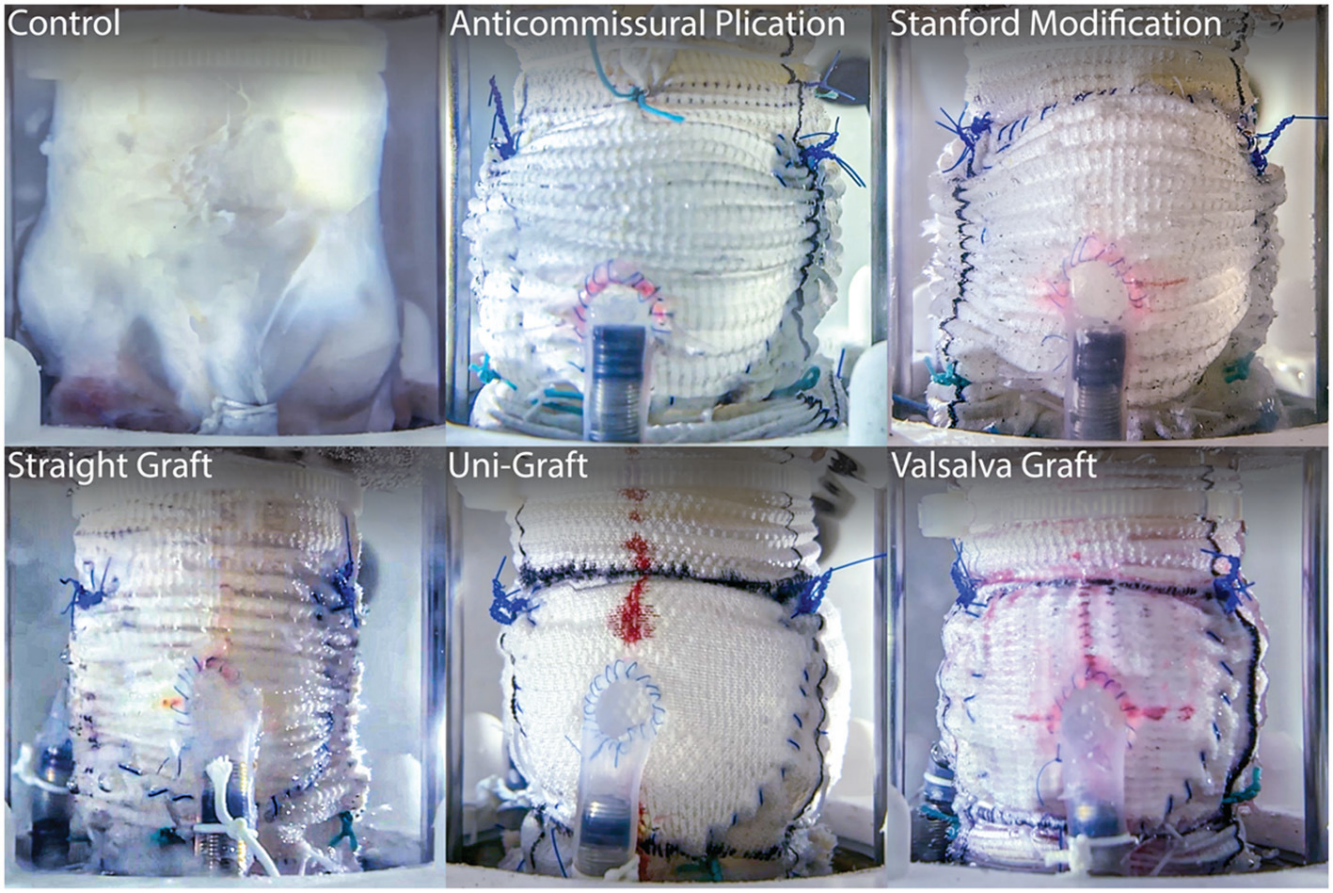
Long-axis view of five clinically used conduit configurations for VSARR, mounted within an *ex vivo* left heart simulator and attached to coronary circulation. *Ex vivo* experimentation allows for highly controlled, direct comparisons of surgical techniques, helping to identify optimal treatments and prostheses. Reprinted from Paulsen et al. (91).

The strength of *ex vivo* experimentation can be seen in a series of AV studies that directly compares treatment techniques for VSARR replacement *via* their biomechanical outcomes (Figure 2). By experimenting with many different clinically used conduit configurations in a heart simulator, researchers were able to identify the subtle hemodynamic differences between repair strategies, bringing quantitative understanding to a topic that was previously, largely based on surgeon preference (91, 92). Specifically, using a combination of pressure, flow, echocardiographic, and high-speed videometric data, the researchers were able to derive a variety of biomechanical parameters including hemodynamics, graft compliance, leaflet kinematics (i.e., mean cusp opening and closing velocity and relative cusp opening and closing force), aortic root distensibility, and pulsed- and continuous-wave Doppler data. From these studies, it was concluded that straight grafts were associated with lower regurgitant fractions and more favorable leaflet mechanics, specifically lower cusp opening and closing velocities and relative forces (*p* ≤ 0.01 for each), most closely recapitulating native aortic root biomechanics. Additionally, work on novel AR models has led to detailed understandings behind disease mechanisms, such as cusp prolapse, bicuspid AV, and root aneurysm, and repairs, such as free margin suspension, free margin plication, and VSARR, which will lead to important insights on optimal repair strategies for AR (93–95). In these studies, while a wide variety of hemodynamic parameters were measured and extracted from pressure and flow data, regurgitation was specifically derived from flow measurements through the AV by integrating flow through the aorta during diastole. Moreover, pulsed- and continuous-wave Doppler, measured *via* echocardiography, provided further biomechanical data regarding regurgitation, peak velocity, peak gradient, and mean gradient. By combining *ex vivo* modeling with clinical guidance and expertise, research groups are leveraging physiologic heart simulators to biomechanically inform surgical practice, guiding optimal treatment strategies and exponentially advancing patient care.

While these technologies have provided incredible insights, *ex vivo* modeling is primarily limited by obstacles in physiologic accuracy and sensing capability. Since this modality uses explanted large animal tissues, fidelity is lost in not only the differences between animal and human hearts but also the inabilities to replicate entire systems and kinematics. An example that highlights this discrepancy is the phase mismatch limitation, which describes the condition where pulsatile piston pumps will initiate expansion of the tissues during systole, when in reality, heart tissue contracts. Regarding sensing capabilities, much work is being done to improve the quantitative metrics measured and derived from *ex vivo* systems. Examples include minimally invasive optical chordal force sensors and coaptation force distribution load sensors (84, 96). These technologies, adapted for heart simulation, can provide deeper insights and improved quantification. Moreover, expanding work has been spearheaded by trailblazing groups that have pushed the edge of capability in the effort to generate greater clinical and physiologic relevance of *ex vivo* simulation.

Two recent studies have pushed the frontiers, leading to a new level of realism for valvular modeling. A significant limitation of current MV simulation is that systems are unable to include the complex motion of the papillary muscles, which have been shown to translate and rotate with every heartbeat *in vivo* (97). These lost interactions crucially affect the motion and forces on the chordae tendineae and leaflets and play an important role for valve function and accurate *ex vivo* modeling. However, in a recent pioneering publication, researchers developed a biomimetic robotic system that captured and replicated this motion *ex vivo* (98). These robots use a six degree-of-freedom motion mechanism that allows for full tracking of the papillary muscle motion in three translation and three rotational axes, showing the effective capability to actuate any physical trajectory within its workspace. This system represents a major advancement in modeling the MV and provides a platform for closer simulation of true physiologic accuracy.

Another monumental achievement in heart simulation presented the development of an organosynthetic, biohybrid heart model that uses custom molded silicone and advanced tissue adhesion capabilities to fabricate a synthetic ventricular myocardium on an explanted porcine heart (99). Researchers implanted pneumatic actuators as the contractile elements in the synthetic myocardium to drive heart function. The model's fidelity was evaluated using echocardiography and 4D MRI and was found to have many geometric and hemodynamic similarities to *in vivo* images. This organosynthetic heart provides an advanced, highly accurate, contractile-based flow generation mechanism that can replace state-of-the-art piston pumps. Specifically, by introducing silicone-embedded contractile elements, this innovative model addresses the inverted phase limitation, one of the largest obstacles of current systems. While integrated sensors can provide hemodynamic data, the most exciting potential is the use of this technology with MRI imaging, as the pneumatically driven actuators allow for a metal-free system. This innovation enables access to a host of MRI-based biomechanical metrics such as high-fidelity 4D flow imaging and analysis, in a geometrically accurate and dynamic left ventricular chamber, leading the way for a new generation of hyper-realistic, *ex vivo* heart simulation technology.

## *In vivo* Modeling

While *in silico* modeling and *ex vivo* modeling provide many valuable insights for developing fundamental and rapid understanding of the heart and valves, large-animal *in vivo* modeling remains the closest among these modalities at studying physiological truth. Large-animal modeling has provided a valuable platform in the valvular biomechanics research ecosystem, and while its primary, traditional use case has been informing surgical technique or device development, pioneering work with implanted, instrumented sensing has shown the potential for using these models to inform the fundamental biomechanics as well.

Early work using large animals primarily centered around ischemic modeling, as coronary vasculature ligation or percutaneous ethanol injection provides straightforward mechanisms for inducing targeted infarction and subsequent ischemia (100–104). In studying ischemic valvular biomechanics, researchers have used these models to analyze a wide variety of phenomena such as ischemic remodeling of MV annular dynamics, surgical techniques, such as posterior leaflet augmentation and chordal cutting, the relationship between ischemic MR and ventricular remodeling, and the effects of annuloplasty ring selection and sizing for ischemic MR repair (102, 103, 105–113). While studies such as these have shown the power of *in vivo* modeling capabilities, there are some key limitations with this line of research. Primarily, working with large-animal models comes with much greater financial costs, limited sample sizes due to administrative humane animal care protocols, and surgical coordination required for developing animal models and conducting *in vivo* experiments, and as such there are few laboratories in the world equipped to execute these kinds of studies. Moreover, the usefulness of *in vivo* experimentation is greatly limited by the number of reliable valvular large-animal disease models, and unfortunately, not many exist (114). However, other works have targeted the expansion of these models including an annular dilation-based, non-ischemic MR model using multiple annular incisions and a mitral leaflet flail model using chordal transection to induce leaflet prolapse and subsequent acute MR (104, 115). Outside of the MV, researchers have worked on a variety of different *in vivo* techniques including pacing-induced tachycardia to generate dilated cardiomyopathy and subsequent valvular regurgitation, valvular lesions to generate aortic constriction, and surgically altered leaflets to generate coaptation failure and AR (114, 116–121).

While much progress is being made to develop new *in vivo* models, biomechanics-focused work aims to generate implantable instrumentation for improved quantification and analysis of underlying phenomena. In a series of studies, researchers developed and utilized a mitral annulus force transducer that measures in-plane radial forces resulting from annular contraction (122). This has led to several lines of experimentation aimed at characterizing mitral annular dynamics in native, pathological, and repair scenarios (123–129). Another instrumented, *in vivo* biomechanics study translates the fiber Bragg grating neochordae, which directly measures chordal forces, for *in vivo* use. These sensors were implanted in a rare ovine case of functional MR, and chordal force profiles were generated for pathological and restored conditions post-annuloplasty. This was the first *in vivo* study to show that mitral ring annuloplasty reduces elevated chordal forces associated with chronic functional MR (130). Publications such as these reveal the potential for implantable, *in vivo* instrumentation to inform valvular biomechanics and surgical repair.

## Comparison of the Modeling Modalities

*In silico* modeling, *ex vivo* modeling, and *in vivo* modeling represent the major capabilities for better understanding and engineering for valvular heart disease. These three modalities comprise the modern ecosystem driving valvular biomechanics research. While each has its own strengths and weaknesses, used in a collaborative, comprehensive manner, modeling technologies can allow for dramatic expansion of this field. The strength of *in silico* modeling lies in the ability to generate numerous new insights and ideas based on a finite element level approach and unique and intuitive quantifications and visualizations, supported by a plethora of data and mathematically derived biomechanical metrics. While these models face challenges, specifically regarding compounding inaccuracies due to homogenized, unifying assumptions that can result in weaker physiologic relevance, the types of questions and hypotheses that can be evaluated *in silico* are distinctively expansive and can provide the basis for deeper investigations. On the other hand, *in vivo* modeling sits on the opposite end of the spectrum in many regards. While these models are almost unparalleled in their physiologic accuracy, they are largely limited by cost, smaller sample sizes, surgical expertise requirements, availability of collaborative interdisciplinary engineering and surgical infrastructure, and the number of reproducible disease models. However, *ex vivo* modeling has the potential to bridge the translational gap for research in this field, leveraging the advantages of both *in silico* and *in vivo* modalities to create an unprecedented level of cost-effective, rapidly iterable, physiologic accuracy. These models have proven to be instrumental to modern research of valvular biomechanics, especially in their ability to immediately inform and improve clinical care.

## Conclusions

The field of heart valve biomechanics is a rapidly expanding, highly clinically relevant area of research that has been driven by crucial *in silico, ex vivo*, and *in vivo* modeling technologies. These modalities represent cutting-edge abilities for generating novel insights regarding native, disease, and repair physiologies, and each has unique advantages and limitations for advancing study in this field. However, leveraged in a collaborative, comprehensive manner, modeling technologies can allow researchers to expand the holistic understanding of heart valves from a wide variety of perspectives, developing critical intuition of these complex systems. Moreover, research in valvular biomechanics has a particularly direct link to clinical translation, as new findings are often immediately and directly manifested and implemented in surgical practice, altering patient outcomes.

In particular, novel *ex vivo* modeling technologies represent an especially promising class of translatable research, providing unique quantitative and qualitative insights on valvular biomechanics. Research that aims to develop new technology adapted for *ex vivo* heart simulation has expanded the capabilities and usefulness of these systems, boosting the clinical and physiologic relevance of this modality while maintaining enough control and measurement ability to generate insightful new quantifications. Overall, the frontiers of this work are being driven by innovative research groups that have used creative, interdisciplinary approaches toward recapitulating *in vivo* physiology, changing the landscape of many related disciplines and positively affecting the lives of patients worldwide.

## Author Contributions

All authors listed have made a substantial, direct, and intellectual contribution to the work and approved it for publication.

## Conflict of Interest

The authors declare that the research was conducted in the absence of any commercial or financial relationships that could be construed as a potential conflict of interest.

## Abbreviations

MV: mitral valve
AV: aortic valve
LV: left ventricle
MR: mitral regurgitation
AR: aortic regurgitation
VSARR: valve-sparing aortic root replacement
MRI: magnetic resonance imaging.
